# *Corchorus olitorius* L. Protects Zebrafish Hair Cells Against Cisplatin-Induced Damage via Antioxidant and Anti-Apoptotic Mechanisms

**DOI:** 10.3390/antiox15060762

**Published:** 2026-06-17

**Authors:** Wei-Sheng Wen, Hsin-Lin Cheng, Zheng-Qi He, Ming-Wei Lee, Yu-Xuan Wu, Tzu-Huan Hung, Shang-Ting Tsai, Po-Hui Wang, Jiann-Jou Yang

**Affiliations:** 1 Institute of Medicine, Chung Shan Medical University, Taichung 40201, Taiwan; 2 Department of Otolaryngology, Chung Shan Medical University Hospital, Taichung 40201, Taiwan; 3 Department of Biomedical Sciences, Chung Shan Medical University, Taichung 40201, Taiwan; 4 Department of Medical Laboratory and Biotechnology, Chung Shan Medical University, Taichung 40201, Taiwan; d880430@csmu.edu.tw; 5 Department of Speech Language Pathology and Audiology, Chung Shan Medical University, Taichung 40201, Taiwan; 6 Department of Clinical Laboratory, Chung Shan Medical University Hospital, Taichung 40201, Taiwan; 7 Crop Genetic Resources and Biotechnology Division, Taiwan Agricultural Research Institute, Taichung 413008, Taiwan; thhong@tari.gov.tw; 8 Department of Applied Chemistry, National Chiayi University, Chiayi City 600355, Taiwan; 9 Department of Obstetrics & Gynecology, Chung Shan Medical University Hospital, Taichung 40201, Taiwan; 10 Department of Medical Research, Chung Shan Medical University Hospital, Taichung 40201, Taiwan

**Keywords:** *Corchorus olitorius* L., transgenic zebrafish, hair cell, cisplatin, antioxidant, ROS

## Abstract

Cisplatin is a widely used platinum-based chemotherapeutic agent that often causes irreversible hair cell loss, leading to hearing impairment. To date, effective strategies for preventing cisplatin-induced ototoxicity remain limited. *Corchorus olitorius* L. (COL) is rich in bioactive phytochemicals with antioxidant and anti-inflammatory properties; however, the protective role of COL stem against cisplatin-induced hearing loss has not been explored. This study aimed to determine whether COL stem extract treatment could mitigate cisplatin-induced hair cell damage in the lateral line system of zebrafish. Herein, we use 7-day post-fertilization (dpf) transgenic zebrafish larvae as a high-throughput screening platform to assessed COL stem extract against cisplatin-induced hair cell injury. Endpoints included mechanotransduction (MET) function, reactive oxygen species (ROS) production, apoptotic and inflammatory responses, and locomotor behavior. Antioxidant capacity and acute toxicity were also evaluated. Pretreatment with COL stem extract preserved hair cell viability, restored MET function, reduced ROS accumulation, upregulated Nrf-2-dependent cytoprotective genes, suppressed apoptosis, and attenuated macrophage infiltration. The recovery of swimming behavior correlated with hair cell protection, confirming the phenotypic relevance. This study demonstrates, for the first time, that COL stem exerts potent otoprotective effects through antioxidative, anti-apoptotic, and anti-inflammatory mechanisms, contributes to maintain mechanosensory function and swimming behavior. The findings support COL stem as a promising candidate for otoprotection and validate zebrafish-based high-throughput screening for novel therapeutic discovery.

## 1. Introduction

Iatrogenic hearing loss represents a prevalent auditory disorder that may manifest throughout lifespan, contributing to impaired quality of life and socio-economic burden on affected patients. The underlying pathology is mainly associated with damage or dysfunction to hair cells, which play a pivotal role in auditory perception [1,2]. The cochlear hair cells of mature mammals possess a limited capacity for spontaneous regeneration following injury, leading to irreversible sensorineural hearing loss. Currently, cisplatin is a representative platinum-containing, cell non-specific chemotherapeutic agent extensively used in clinical oncology for the treatment of malignant diseases, including bladder, head and neck, cervical, lung and breast cancers [3,4,5,6]. Notably, cisplatin-induced ototoxicity, with an estimated incidence of approximately 50%, represents a serious dose-limiting side effect that lacks an effective preventive strategy, thereby restricting its clinical application [7,8]. The mechanistic basis of cisplatin-induced ototoxicity remains to be fully elucidated. Accumulating evidence suggests that cisplatin-induced oxidative stress in the inner ear contributes to the pathogenesis of ototoxicity and is primarily mediated by reactive oxygen species (ROS) production [9]. Therefore, mitigating ROS accumulation and modulating antioxidant pathway may provide a promising strategy for preventing cisplatin-induced hair cell loss.

Jute mallow (*Corchorus olitorius* L., COL), belonging to the Malvaceae family, is an annual plant primarily cultivated in Asia, Africa, and Middle East for the production of commercial bast fiber [10,11,12]. The tender stems and leaves are used as edible vegetables with recognized application in folk herbal medicine in several areas such as Egypt and Taiwan [13,14,15]. Existing evidence indicates that different parts of COL, such as root, leaf, seed, and stem are rich in bioactive components, which include β-carotene, coumarins, ionones glucosides, phenolic acid and flavonoids, and possesses therapeutic effects for fever, heatstroke, constipation, diarrhea, and cystitis [16,17,18]. Research on COL-derived dicaffeoylquinic acid exhibited a neuroportective effect through targeting the pro-inflammatory mediators in neuroinflammatory mouse model [19,20]. Furthermore, Zakaria et al. evaluated the effects of COL on nociceptive responses using the hot-plate and abdominal constriction tests in ICR mice [21]. The antinociceptive profile showed a conspicuous increased of latency time for discomfort response in both tests, indicating significant antinociceptive and anti-inflammatory properties. These pharmacological activities of COL are mainly attributed to its bioactive phytochemicals, which have been investigated for their antioxidant properties. However, the available literature on the beneficial effects of COL is limited and warrants further investigation.

Bioactive phytochemicals obtained from natural sources have demonstrated significant antioxidant activity, which enhanced endogenous antioxidant defense mechanisms and contributed to disease prevention. To date, the protective role of COL against cisplatin-induced ototoxicity has not yet been explored. Our central hypothesis is that COL extract pretreatment can attenuate cisplatin-induced hair cell loss by mediating cellular pathways associated with antioxidant defense and anti-inflammatory responses. Proving this hypothesis would facilitate the development of an alternative therapeutic strategy for protecting auditory function against cisplatin toxicity.

## 2. Materials and Methods

### 2.1. COL Stem Extraction Preparation

Fresh stem part of COL was collected from the local area of Taichung city (Taiwan). The freeze-dried COL stem was ground into powder. To maximize target compound yield and align with the traditional culinary usage in Taiwan (jute soup), the powder was extracted with hot water at 90 °C (1:25 ratio, *w*/*v*) for 120 min. Specifically, continuous ultrasonication (60 kHz; ES-600N, Taiwan Supercritical Technology Co., Ltd., Changhua, Taiwan) was applied to expedite the extraction rate and minimize potential thermal degradation. Following the extraction, the mixture was filtered through an 80-mesh filter bag to separate the crude extract from the insoluble stem residues. The resulting filtrate was concentrated to a quarter of its initial volume and freeze-dried using a lyophilizer (FD-5020, PANCHUM Scientific Corporation, Kaohsiung, Taiwan) to avoid further metabolite degradation and obtain the final powder. The powder was subsequently stored at −20 °C until use.

### 2.2. Total Polyphenol and Flavonoid Content Determination

Phytochemical analysis was carried out using a colorimetric approach based on previous description [9]. For Folin–Ciocalteu method, a reaction mixture was prepared by combining 10 μL of COL stem extracts with 200 μL of distilled water and 100 μL of Folin–Ciocalteu reagent. After thorough homogenization, 500 μL of Na_2_CO_3_ solution (10% *w*/*v*) was added to the mixture. The reaction was then allowed to processed for 20 min at room temperature in the dark. Subsequently, the absorbance was measured at 736 nm using a spectrophotometer (SpectraMax M5, Molecular Devices, San Jose, CA, USA). The total phenolic content was determined using a gallic acid standard curve (y = 0.481x − 0.0213, R^2^ = 0.9966) and the results were expressed as mg of gallic acid equivalent (GAE) per g of dry weight (DW). For aluminum chloride method, a reaction mixture consisting of 10 μL of COL stem extracts, 500 μL of distilled water, and 30 μL of NaNO_2_ (5% *w*/*v*) was thoroughly homogenized. After incubating for 6 min at room temperature, 60 μL of AlCl_3_ (5% *w*/*v*) solution was added, mixed completely, and allowed stand for an additional 5 min. Afterward, 200 μL of NaOH (1 M) solution and 110 μL of distilled water were added into the mixture. The final absorbance was subsequently measure at 510 nm using a spectrophotometer. The total flavonoids content was determined using a catechin standard curve (y = 0.897x + 0.0061, R^2^ = 0.9996) and results were expressed as mg of catechin equivalent (CE) per g of dry weight (DW).

### 2.3. Determination of Antioxidant Activity

Antioxidant capacity of COL stem extract was analyzed using the 2,2-Diphenyl-2-picrylhydrazyl (DPPH) radical scavenging assay and Trolox equivalent antioxidant capacity (TEAC) assay based on previous description [9]. For the DPPH assay, 50 μL of COL stem extracts was mixed with 200 μL of 1 mM DPPH^•^ working solution prepared in absolute methanol (Anhydrous ≥ 99.9%. Avantor, Inc., Radnor, PA, USA). The mixture was incubated in the dark for 30 min at room temperature, after which the absorbance (517 nm) was measure using a spectrophotometer. A standard curve in the range of 1–10 mg/mL was prepared for gallic acid and the scavenged 50% (IC_50_) of DPPH^•^ radicals were expressed as mg of gallic acid equivalent (GAE) per g of dry weight (DW). For TEAC assay, ABTS^+^ working solution was prepared by mixing (1:1:1 *v*/*v*/*v*) peroxidase (4.4 unit/mL), ABTS (100 μM), and H_2_O_2_ (50 μM) solution. The COL stem extracts of 30 μL was mixed with 970 μL of ABTS^+^ working solution and kept in the dark for 1 min at room temperature. Finally, the absorbance at 517 nm was measure using a spectrophotometer. A standard curve in the range of 1–10 mg/mL was prepared for Trolox and IC_50_ of ABTS^+^ radical were expressed as mg of Trolox equivalent (TEAC) per g of dry weight (DW).

### 2.4. Ultra-Performance Liquid Chromatograph–High-Resolution Mass Spectrometry (UPLC-HRMS) Analysis

The chemical profiling was carried out following a previously described method [22], using a Thermo Ultimate 3000 UHPLC system interfaced with an Orbitrap Fusion Tribrid mass spectrometer (Thermo Fisher Scientific, Waltham, MA, USA). Chromatographic separation was achieved on an ACQUITY UPLC HSS T3 column (2.1 mm × 100 mm, 1.8 μm; Waters, Milford, MA, USA) and maintained at a constant temperature of 40 °C. The binary mobile phase consisted of water with 0.1% formic acid (solvent A) and acetonitrile with 0.1% formic acid (solvent B). The flow rate was set at 0.4 mL/min with the gradient elution programmed as follows: an initial hold at 5% B for 2 min; a linear increase from 5% to 20% B (2–15 min); a ramp to 80% B (15–22 min); a further increase to 99.9% B (22–27 min); an isocratic hold at 99.9% B (27–28.5 min); a rapid drop to 5% B (28.5–28.6 min); and a final re-equilibration at 5% B (28.6–30 min). Mass spectrometry detection was performed in negative electrospray ionization (ESI−) mode. The ion source parameters were optimized as follows: spray voltage at 2.5 kV, ion transfer tube temperature at 325 °C, and vaporizer temperature at 350 °C. The flow rates for sheath gas, auxiliary gas, and sweep gas were set to 50, 10, and 3 arbitrary units (arb), respectively. Full MS scans were acquired over a mass range of *m*/*z* 100–1200. For tandem mass spectrometry (MS/MS) data acquisition, higher-energy collisional dissociation (HCD) was applied using a stepped collision energy of 35 ± 15%. To identify the metabolites, their retention times (RTs), accurately measured *m*/*z* values, and MS/MS fragmentation profiles were systematically compared with reference data available in public databases such as GNPS, MassBank, and PlaSMA.

### 2.5. Animals

The (*pvalb3b: Tag*GFP) transgenic zebrafish line specifically expressed green fluorescence protein (GFP) in the hair cells of lateral line neuromasts [23,24]. All fish were maintained in a circulatory system and standard conditions at university’s laboratory animal center. Embryos were obtained from the mating of adult fish and raised at 28 °C in fresh egg water (60 μg/mL sea salt in distilled water) with 14/10 h light–dark cycle. Zebrafish larvae 7 days post-fertilization (dpf) were used in all experiments. All experiments and protocols involving zebrafish were performed in accordance with the guidelines of the Animal Care and Use Committee of Chung Shun Medical University (protocol code: 115082. Date: 15 January 2026).

### 2.6. Animal Treatments

A pretreatment method was performed to evaluate the otoprotective effects of COL stem extracts on cusplatin-induced hair cell damage. Transgenic zebrafish larvae at 7-dpf were exposed to COL stem extracts at concentrations of 0.5, 1.0, 1.5, 2.0, 2.5, and 3.0 mg/mL for 1 h. Larvae were subsequently co-exposed to cisplaitn (Sigma-Aldrich, St. Louis, MO, USA) for 1 h through the addition of cisplatin stock solution to a final concentration of 250 μM. At the end of the exposure period, the live larvae and hair cells within the neuromasts (Middle: MI, otic: O, occipital: OC, and posterior lateral line-1, -3, -4: PLL-1, -3, -4) of lateral line system were observed and quantified using an inverted phase contrast fluorescence microscope (Olympus CKX53, Tokyo, Japan). At the end of the experiments, all remaining larvae were humanely culled through an overdose of tricaine methanesulfonate (MS-222) followed by rapid freezing.

### 2.7. FM4-64dye Labeling

To evaluate the functionality of hair cells in the lateral line system, FM4-64 dye was employed to label surviving live hair cells in neuromasts. After the pretreatment procedures, 7-dpf larvae were immersed in FM4-64 (N-(3-Triethylammoniumpropyl)-4-(6-(4-(Diethylamino) Phenyl) Hexatrienyl) Pyridinium Dibromide) (Thermo Scientific, Rockford, IL, USA) dye (50 μM) for 15 min in the dark, followed by rinsing three times in PBS to terminate staining reaction. The larvae were anesthetized in 0.01% tricaine methanesulfonate (MS-222, Sigma-Aldrich, St. Louis, MO, USA) and then immobilized with 0.5% low-melting agarose solution on a slide under inverted phase contrast fluorescence microscope.

### 2.8. TUNEL Assay

Apoptotic cells in the neuromasts was identified through the terminal deoxynucleotidyl transferase dUTP nick-end labeling (TUNEL) method using an in situ cell detection kit (Roche Diagnostics GmbH, Mannheim, Germany) following the manufacturer’s instruction. After the pretreatment procedure, the larvae were fixed for 16 h in freshly prepared 4% paraformaldehyde at room temperature. Thereafter, the fixed larvae were washed three times with PBST (1× PBS/0.2% Tween-20) and permeabilized with acetone (T.J Baker Inc., Phillipsburg, NJ, USA) for 1 h. After permeabilization, the larvae were washed in PBS and stained in a mixture of label solution and enzyme solution in the ratio of 1 to 9. The total number of live hair cell and TUNEL-positive cells in the anterior region of lateral line system were photographed using inverted phase contrast fluorescence microscope.

### 2.9. Macrophage Neutral Red (NR) Staining

Neutral red (NR) uptake method was conducted for in vivo detection of macrophages due to the phagocytic ability. To evaluate macrophages localized in the lateral line system, the larvae form each experimental group were respectively stained in a 3 cm Petri dish containing 2 μg/mL of NR (0.33%, Sigma-Aldrich, St. Louis, MO, USA) dye solution. After 90 min incubation, the stained larvae were cleared in fresh egg water and photographed using inverted phase contrast fluorescence microscope.

### 2.10. Reactive Oxygen Species (ROS) Detection

The ROS level was evaluated using CellROX Orange reagent (Thermo Scientific, Rockford, IL, USA) following the manufacturer’s instruction. Briefly, the larvae were stained for 30 min in a working solution containing 5 μM of CellROX Orange dye in a well at 37 °C. After removing the fluorescence dye, the stained larvae were washed three times with PBS, and ROS production in hair cells was then observed and photographed using inverted phase contrast fluorescence microscope.

### 2.11. Quantitative Real-Time PCR (qPCR) Analysis

Total RNA was isolated from 15 zebrafish larvae using EasyPrep Total RNA kit (QIAGEN Ltd., Hilden, Germany) and a total of 2 μg RNA was reverse-transcribed into cDNA by using RevertAid First Strand cDNA Synthesis Kit (Thermo Scientific, Rockford, IL, USA) following the manufacturer’s instruction. The q-PCR analysis was performed on a Real-Time PCR system (Applied Biosystems Inc., Waltham, MA, USA). The primers were: *gpx*-F: 5′-TCCGTCCTGTGTTTTGGGCT-3′, *gpx*-R: 5′-AGGCTTGAGGATCAGCCGTC-3′; *nrf-2*-F: 5′-ATGGAGCTGCTGTCACTCCC-3′, *nrf-2*-R: 5′-TTCTGTTTGAGCCGAGCCGA-3′; *sod1*-F: 5′-GAAGTGACCGGCACCGTCTA-3′, *sod1*-R: 5′-TGCACTGATGCAGCCGTTTG-3′; *cat*-F: 5′-CGATCGCTGTCCGCTTTTCC-3′, *cat*-R: 5′-ATCCCAGTTGCCCTCATCGG-3′; *gstp1*-F: 5′-TGCACTGCAGACTAGGAGCA-3′, *gstp1*-R: 5′-GTGGCTTTCAAGTCGCCCTT-3′; *sod2*-F: 5′-GCGTGTGCTAACCAAGACCC-3′, *sod2*-R: 5′-TTGGCAGCTTGGAAACGCTC-3′; *β-actin*-F: 5′-ACGGTATTGTCTGGTGGTAC-3′, *β-actin*-R: 5′-TACTCCTGC TTGCTAATCC-3′. The mRNA expression levels were normalized to the level of β-actin and were determined based on the comparative *Ct* method.

### 2.12. Behavioral Assay and Analysis

Behavioral analysis was conducted using a closed chamber system and tracking software (EthoVision XT, version 11.5, Noldus, Wageningen, The Netherlands) for comprehensively monitoring transgenic zebrafish larvae locomotors activity, according to our previously established method [25]. The 7-dpf transgenic zebrafish larvae were individually placed in a 24-well plate and their avoidance behaviors in response to tapping stimulus (intensity 8), including distance moved, swimming velocity, turn angle, and rotation frequency, were recorded. The experimental protocol involved a total of 75 s tracking times. After the tapping stimulus was applied, the locomotor trajectories of the transgenic larvae within open arena were continuously monitored using tracking software.

### 2.13. Statistical Analysis

All experimental data were expressed as the mean ± standard deviation (SD) or standard error (SEM). The statistical analysis was performed with SPSS software V 22.0 (SPSS Inc., Chicago, IL, USA) using one-way ANOVA followed by Duncan’s post hoc test. Statistical differences were determined at *p* values under 0.05.

## 3. Results

### 3.1. Evaluation of Phytochemical Contents and Antioxidant Activity of COL Stem

As shown in Table 1, the total phenolic and flavonoids contents of COL stem extract were identified as 7.43 ± 0.68 mg of gallic acid equivalent per g of dry weight (mg GAE/g DW) and 14.75 ± 2.52 mg GAE/g DW, respectively. Additionally, the radical scavenging capacity of COL stem extract further corroborates the biological activity of phenolic and flavonoids contents. Moreover, COL stem extract exhibited strong antioxidant activities with 6.17 ± 0.35 mg/mL mg GAE/g DW for DPPH method and 142.35 ± 5.74 mg TEAC/g DW in ABTS method, respectively (Table 2).

A lower IC_50_ value correspond to higher radical scavenging potential, suggesting a stronger antioxidant capacity, with IC_50_ values of 6.19 ± 0.00 mg/mL (DPPH) and 13.92 ± 0.00 mg/mL (ABTS).

**Table 1:** Total phenolic and flavonoid contents of COL stem.

Extract	Total Phenolic Content (mg GAE/g DW)	Total Flavonoid Content (mg CE/g DW)
Water extract	7.43 ± 0.68	14.75 ± 2.52

Values are present as mean ± standard deviation (SD); GAE—gallic acid equivalent; CE—catechin equivalent.

**Table 2:** Antioxidant capacity of COL stem from DPPH and ABTS assay.

Extract	DPPH		ABTS	
	IC_50_ (mg/mL)	Antioxidant Activity (mg GAE/g DW)	IC_50_ (mg/mL)	Antioxidant Activity (mg TEAC/g DW)
Water extract	6.19 ± 0.00	6.17 ± 0.35	13.92 ± 0.00	142.35 ± 5.74

Values are present as mean ± standard deviation (SD); GAE—gallic acid equivalent; TEAC—Trolox equivalent antioxidant capacity.

### 3.2. Chemical Profiling of COL Stem Extract

**Figure 1:**
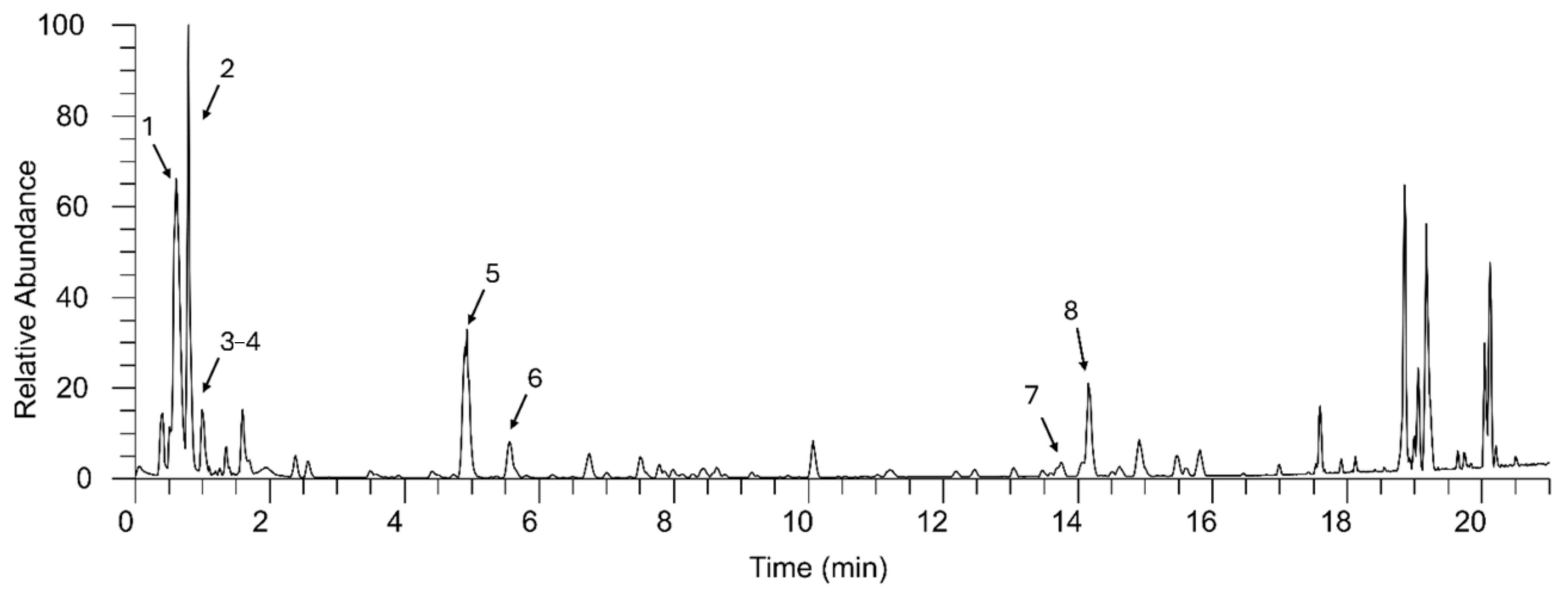
Representative base peak chromatogram of COL stem extract.

The chemical composition of the COL stem extract was analyzed using UPLC-HRMS in a negative ESI mode. By comparing exact masses and MS/MS fragmentation patterns with public databases, eight major metabolites were tentatively identified (Figure 1 and Table 3). The identified compounds include organic acids (malic acid, citric acid, and succinic acid), a nucleoside (guanosine), a dicarboxylic acid (azelaic acid), and phenolic compounds (chlorogenic acid, cryptochlorogenic acid, and cynarin).

### 3.3. COL Stem Protected Against Cisplatin-Induced Hair Cell Damage in Transgenic Zebrafish

**Figure 2:**
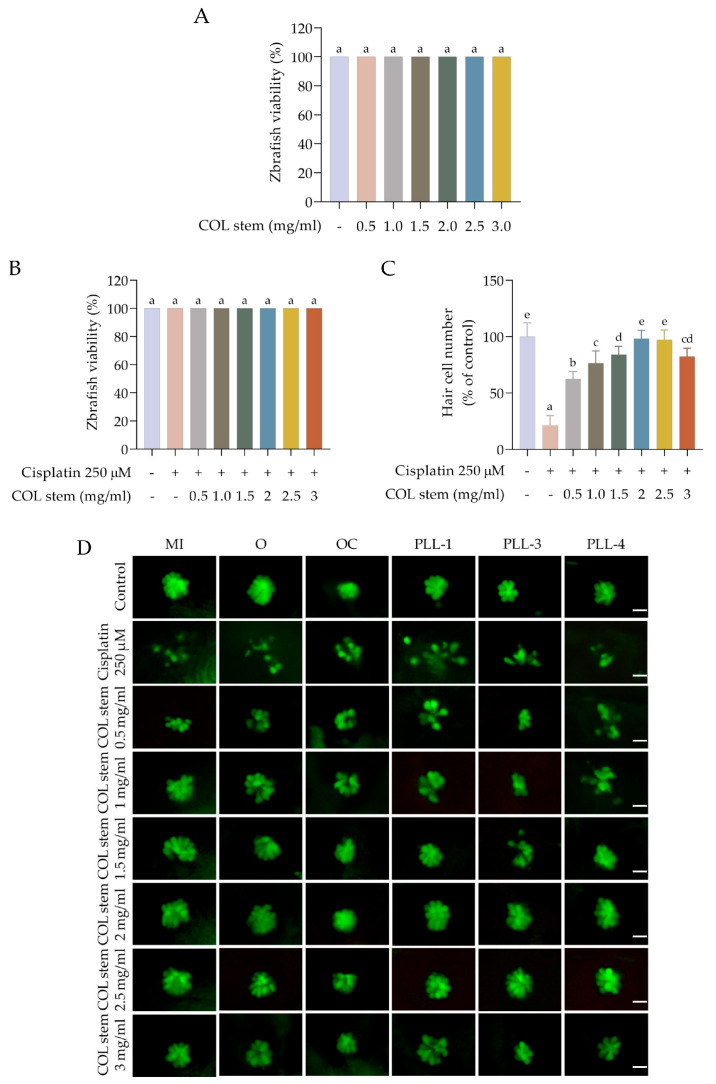
Protective effects of COL stem extract on cisplatin-induced hair cell loss in transgenic zebrafish. The 7-dpf zebrafish larvae were pretreated with COL stem extract (0.5, 1.0, 1.5, 2.0, 2.5, and 3.0 mg/mL) for 1 h, followed by co-treatment with cisplatin 250 μM for 1 h. (**A**) Zebrafish viability showing no significant acute toxicity in COL-stem-extract-treated groups compared with control. (**B**) Quantification of zebrafish viability among experimental groups and (**C**) hair cell counts shows that COL stem extract significantly prevented cisplatin-induced hair cell loss. (**D**) Representative images of hair cell within MI, O, OC, PLL-1, PLL-3, and PLL-4 neuromasts in each experimental group. Scale bar: 10 μm. Data are presented as mean ± SD (n = 30). Different letters indicate significant differences between groups at *p* < 0.05, based on one-way ANOVA with post hoc Duncan’s multiple range tests.

To assess whether COL stem extract protects against cisplatin-induced damage in lateral line hair cells, a series of dose–response titrations was conducted to determine its otoprotective ability. No statistical difference was observed in zebrafish viability among COL-stem-extract-treated groups, suggesting no signs of cytotoxicity (Figure 2A). Pretreatment with COL stem extract at 0.5, 1.0, 1.5, 2.0, 2.5, and 3.0 mg/mL for 1 h followed by cisplatin co-treatment for 1 h significantly increased number of hair cells, when compared with the cisplatin group (Figure 2B–D). Additionally, the optimal otoprotective concentration of COL stem extract was identified as 2.0 mg/mL (98.30%) in 7-dpf zebrafish. Thus, COL stem concentrations of 0.5, 1.0 and 2.0 mg/mL were used in the subsequent experiments to evaluate its protective roles.

### 3.4. COL Stem Protected Against Cisplatin-Induced Apoptosis and Mechanotransduction Impairment in Transgenic Zebrafish

**Figure 3:**
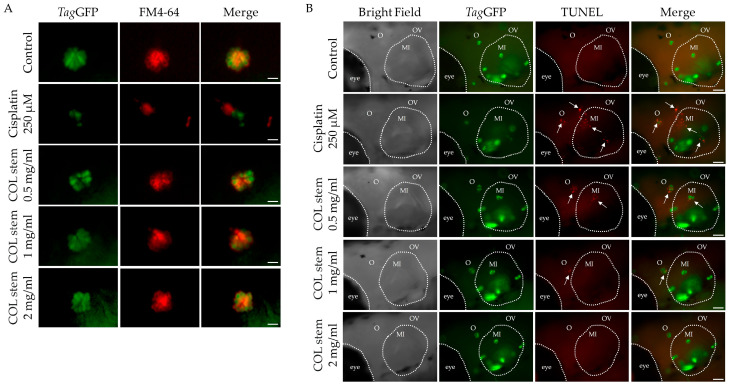
Effects of col stem extract on mechanotransduction integrity and apoptotic response in cisplatin-treated transgenic zebrafish. The 7-dpf transgenic zebrafish larvae were stained with FM4-64 to visualize hair cell membrane integrity and TUNEL assay to detect apoptotic cells. (**A**) Representative FM4-64 images show reduced membrane labeling and cell internalization following cisplatin treatment, which was restored by COL stem extract pretreatment, paralleling the trend in hair cell viability. Scale bar: 10 μm. (**B**) TUNEL staining demonstrates a marked increase in apoptotic cells in the anterior region of lateral line neuromasts after cisplatin exposure, whereas pretreatment with COL stem extract resulted in a reduction in TUNEL-positive cells (red spot, indicated by white arrow). OV, oval window; O, occipital; MI, otic. Scale bar: 50 μm.

The transgenic zebrafish larvae stained with FM4-64 dye shows a loose of membrane labeling and reduced cell internalization after cisplaitn treatment. COL stem extract pretreatment revealed that FM4-64-lebeled membrane increased, similar to the trend of hair cell viability (Figure 3A). In addition, the resulting images also revealed that the TUNEL-positive fluorescent particles in cisplatin group greatly increased in anterior region of lateral line, as compared to the untreated control. However, a subsequent dose-related reduction in TUNEL-positive cells was observed (Figure 3B).

### 3.5. COL Stem Attenuates Cisplatin-Inudced Oxidative Stress and Inflammatory Cell Infiltration in Transgenic Zebrafish

**Figure 4:**
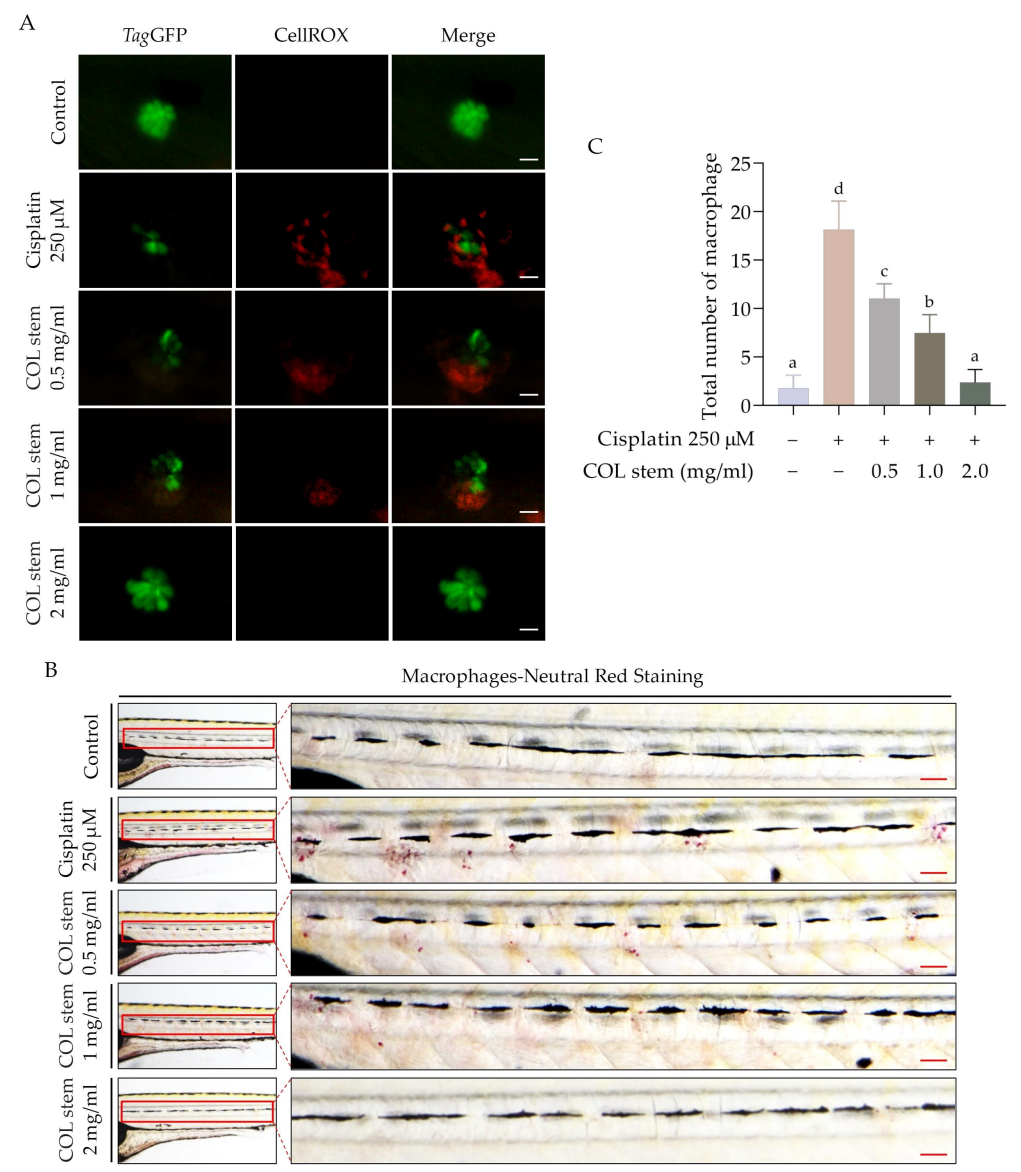
Effect of COL stem extract on cisplatin-induced oxidative stress and inflammatory responses in transgenic zebrafish. (**A**) CellROX staining illustrating ROS production in neuromasts. Cisplatin induced widespread ROS accumulation, while pretreatment with COL stem extract attenuated oxidative stress. Scale bar: 10 μm. (**B**,**C**) Neutral red (NR) staining showing macrophage accumulation (purple particles) in lateral line neuromasts. Cisplatin treatment markedly increased macrophage infiltration compared with control, whereas COL stem extract reduced macrophage accumulation in a dose-dependent manner. Data are presented as mean ± SD (n = 30). Different letters indicate significant differences between groups at *p* < 0.05, based on one-way ANOVA with post hoc Duncan’s multiple range tests. Scale bar: 50 μm.

Next, we assess cisplatin-triggered ROS generation and inflammatory response that leads to macrophages accumulation. NR staining was performed to visualize the macrophages and reflected the inflammatory response in zebrafish larvae. Compared with control group, there was a significant purple particle accumulated in the lateral line neuromasts of zebrafish larvae in the cisplatin group, indicating local inflammation and macrophages infiltration. However, the cisplatin-induced macrophage accumulation in the lateral line neuromasts was significantly decreased in the COL-stem-extract-treated group in a dose-dependent manner (Figure 4A). Meanwhile, cisplatin treatment triggered a substantial and diffuse CellROX signal throughout the neuromasts, indicating elevated oxidative stress compared with the control group, which was consistent with the trend observed in NR staining. However, the pretreatment with the COL stem extract effectively attenuated excessive ROS production (Figure 4B,C).

### 3.6. COL Stem Restores Cisplatin-Inudced Depletion of Antioxidant Status in Transgenic Zebrafish

**Figure 5:**
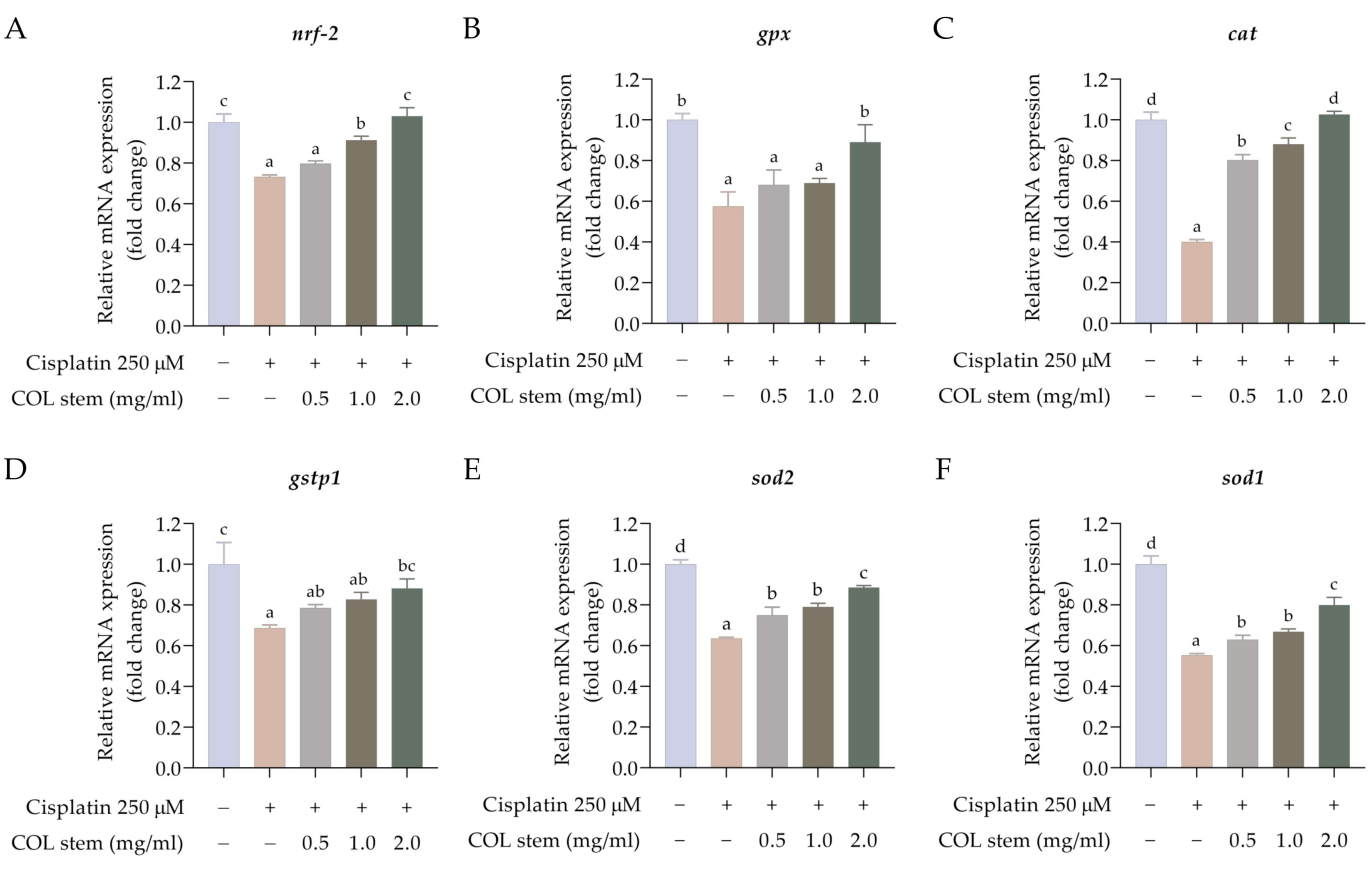
Effect of COL stem extract on antioxidant gene expression in cisplatin-induced hair cell damage. The 7-dpf zebrafish larvae were pretreated with COL stem extract (0.5, 1.0, and 2.0 mg/mL) for 1 h, followed by cisplatin exposure for 1 h. Quantitative PCR (q-PCR) analysis was performed to assess the gene expression of antioxidant-related enzymes. Pretreatment with COL stem extract reversed the cisplatin-induced downregulation of (**A**) *nrf-2*, (**B**) *gpx*, (**C**) *cat*, (**D**) *gstp1*, (**E**) *sod2*, and (**F**) *sod1* in a dose-dependent manner, supporting its protective role against cisplatin ototoxicity. Data are presented as mean ± SD. Different letters indicate significant differences between groups at *p* < 0.05, based on one-way ANOVA with post hoc Duncan’s multiple range tests. *nrf-2*: nuclear factor erythroid 2-related factor 2, *gpx*: glutathione peroxidase 1, *cat*: catalase, gstp1: glutathione s-transferase pi 1, *sod2*: superoxide dismutase type 2, *sod1*: superoxide dismutase type 1.

Next, we assessed gene expression relevant to antioxidant metabolic enzymes. The q-PCR revealed that *sod1*, *sod2*, *nrf-2*, *gstp1*, *gpx*, and *cat* expression were significantly decreased after cisplatin treatment, which further corroborated the role of ROS-mediated apoptosis in hair cells (Figure 5). Pretreatment with COL stem extract reversed the downregulated expression of those genes induced by cisplatin-mediated damage. These comprehensive finding indicated the protective role of COL stem extract on cisplatin ototoxicity in lateral line hair cells.

### 3.7. COL Stem Attenuates Cisplatin-Inudced Deficits in Zebrafish Swimming Behavior

**Figure 6:**
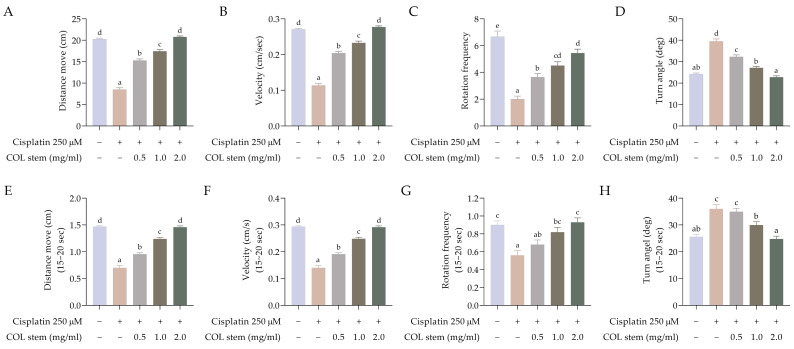
Effect of COL stem extract on cisplatin-induced locomotor dysfunction in transgenic zebrafish. Locomotor responses were recorded following a tapping stimulus, and behavioral parameters including (**A**) distance moved, (**B**) velocity, (**C**) rotation frequency, and (**D**) turn angle were analyzed over the whole testing period. Escape responses during the 5 s interval within 15–20 s were also analyzed (**E**–**H**), showing trends consistent with the overall behavioral patterns (**A**–**D**). Data are presented as mean ± SEM. Different letters indicate significant differences between groups at *p* < 0.05, based on one-way ANOVA with post hoc Duncan’s multiple range tests.

Subsequently, we assessed the whole-time behavioral responses to compare distance move, velocity, rotation frequency, and turn angle of zebrafish larvae between each group after a tapping stimulus. The cisplatin treatment induced impaired swimming behavior in zebrafish larvae, characterized by a significantly decreased in distance moved, velocity, and rotation frequency, along with an increase in turn angle compared with the control group. In contrast, pretreatment with COL stem extract gradually restored these behavioral changes in a dose-dependent manner (Figure 6A–D). Moreover, the analysis of the 5 s escape response window (15–20 s post-stimulus) exhibited a trend consistent with the whole-time behavioral analysis (Figure 6E–H).

## 4. Discussion

Previous studies have reported that different parts of COL are rich in bioactive phytochemicals, which contributed to its antioxidant and anti-inflammatory properties, highlighting its critical roles in the treatment of inflammatory-associated diseases [20,26,27]. However, the potential impact of COL stem on sensorineural hearing loss has not yet been explored. To the best of our knowledge, the present work is the first to provide comprehensive in vivo evidence elucidating the mechanistic pathway underlying COL-stem-mediated protection against cisplatin ototoxicity. Here, we implemented a high-throughput rapid screening strategy to discover and characterize novel agent against cisplatin-induced hair cell loss [23]. Multiple functional endpoints, including mechanotransduction machinery, apoptotic response, redox homeostasis, and behavioral outcomes, were systemically investigated using 7-dpf zebrafish larvae as a screening platform for cisplatin-mediated ototoxicity.

Despite the application of ethnomedicinal uses, limited information is available regarding the COL-stem-derived active constituents, biological functions, and safety profiles. The existing literature has reported preliminary description of phytoremediation, morphological, and phytotherapeutic properties of COL stem [13,28,29,30]. These findings are critical for establishing the scientific significance of the stem and other plant parts of COL. Regarding the chemical constituents, our UPLC-HRMS analysis established that the COL stem extract is a rich source of phenolic compounds, most notably chlorogenic acid, cryptochlorogenic acid, and cynarin (Table 3). According to the literature, β-amyrin, palmitic acid, and stearic acid constitute the major components of the stem part of COL [31]. Previous phytochemical screenings of C. olitorius stems have demonstrated a rich yield of polyphenol compounds, with prominent profiles of ferulic acid, chlorogenic acid, catechin, pyrogallol, and ellagic acid [32]. Various phenolic acids, occurring as conjugated forms through glycosylation and esterification, have also been identified in the stem of COL [33]. In addition, phyto-analysis of the methanolic extract from different parts (seeds, roots, stems and leaves) of COL revealed a phytosterol, which was identified using spectroscopic methods [31]. It has been noted that COL contained phytochemicals produced during its phenological growth stages. Ashok and colleagues reported the average phenolic and flavonoid contents of COL stem, while our findings demonstrated higher levels of total phenol and flavonoids in ultrasonic water extract of COL stem, as depicted in Table 1 [17]. Similarly to the report by Biswas et al., the DPPH and ABTS radical scavenging methods revealed significant antioxidant activity of COL stem extract, which might be attributed to those phytochemicals, suggesting a potential role in reducing ROS-mediated apoptosis (Table 2 and Table 3) [16]. Additionally, an acute toxicity assessment was conducted to provide preliminary indicators of potential toxicological effects. The COL stem extract at doses up to 3 mg/mL was used in the present study, as no overt signs of toxicity or mortality were observed.

It has been documented that cisplatin ototoxicity leads to the loss of hair cell viability. Since no significant toxic effects was detected in our in vivo zebrafish model, COL stem extract exhibited an optimal protective concentration at 2 mg/mL, significantly improving hair cell viability to levels close to those of the control group (98.3%). Such otoprotective agents have also been discussed in the literature. Pretreatment with Korean red ginseng (2.5 mg/mL) significantly improved HEI-OC1 auditory cell viability by reducing cisplatin-induced ROS overproduction and suppressing apoptosis-related signaling pathway [34]. In vivo evidence demonstrated that low dose of apocynin (250 μM) significantly attenuated cisplatin-induced hair cell loss in 5-dpf zebrafish larvae. The protective actions were also associated with diminished both ROS generation and caspase-3 enzymatic activity HEI-OC1 cells [35]. Intriguingly, Thomas et al. evaluated 10,000 otoprotective candidates using a screening method in 5-dpf zebrafish larvae and subsequently identified two of the most promising molecules, which display dose-dependent otoprotective effects by inhibiting cisplaitn uptake [36]. Collectively, the aforementioned studies further support our high-throughput screening method as an effective platform for identifying protective agents against iatrogenic hearing loss.

In mechanosensory hair cells, functional mechanotransducer (MET) channels are necessary for cisplatin ototoxicity [37,38,39]. Zebrafish lateral line hair cells share stereociliary MET processes with mammalian inner ear hair cells, like their mammalian counterparts, are susceptible to cisplatin, which rapidly accumulates in the cytoplasm and activates caspase-dependent apoptotic signaling [40,41]. To determine whether COL stem extract acts as a pharmacological blocker of the hair cell MET channel and thereby preserves cell viability, FM4-64 dye was used to assess the functional integrity of MET. The exposure to cisplatin resulted in hair cell depletion per neuromasts, accompanied by a failure to accumulate FM4-64 fluorescent labeling in hair cells. Remarkably, the COL stem extract pretreatment effectively restored the diminished dye uptake in hair cells, combined with improved cell body optical clarity, reaching near-control levels at 2 mg/mL. This observation is consistent with previous studies showing that fasudil and naringin prevented cisplatin-induced hair cells damage in zebrafish models, as evidenced by increasing FM1-43 fluorescence intensity and preserved MET function [42,43]. Excessive ROS generation directly impaired mitochondrial biogenesis, caused DNA damage, and ultimately triggered apoptotic hair cell death [44]. To validate the mechanistic hypothesis, we performed experiments involving cellular stress and apoptotic markers. The results of CellROX stain and TUNEL assay revealed that pretreatment of COL stem extract resulted in an inhibition of ROS-mediated apoptotic cascade, as demonstrated by decreasing CellROX-positive orange labeling and TUNEL-positive signals in neuromasts. Furthermore, accumulating studies suggest that inflammation acts as an upstream initiating factor in cisplatin-induced ototoxicity and is mainly mediated by immune response involving phagocyte production, predominantly macrophages [45,46]. The attenuation of macrophage accumulation in lateral line neuromasts through COL stem extract pretreatment closely paralleled that of reduced ROS levels and apoptotic response, which verified a pivotal role of ROS-driven inflammatory and apoptotic cascade. The pharmacological profiling of cryptochlorogenic acid highlighted its capacity to suppress inflammatory symptoms in RAW 264.7 macrophages by dose-dependently inhibiting the production of nitric oxide (NO), TNF-α, and IL-6, while simultaneously downregulating iNOS and COX-2 expressions [47]. Evidence from Lin et al. demonstrated that chlorogenic acid attenuated serum TNF-α, IL-1β, MDA, and ROS levels, which is consistent with histopathological findings showing the protection of outer hair cells and significant improvements in auditory startle responses in an ototoxic rat model [48]. An earlier study by Borse et al. revealed that oral gavage of epigallocatechin-3-gallate attenuated cisplaitn-induced cochleotoxicity by reducing oxidative stress, upregulating STAT3/Bcl-2/Bcl-xL pathway, consistent with in vitro finding showing suppression of ROS generation and TUNEL-positive signals in cochlear tissue [49]. Transcriptomic analyses of spiral ganglia following kanamycin-induced hair cell loss revealed early activation of immune-inflammatory pathways and macrophage infiltration, with subsequently spiral ganglion neuron apoptosis, whereas heterogeneous anti-inflammatory intervention significantly attenuated macrophage activation and chronic spiral ganglion neuron degeneration [50]. In mouse and HEI-OC1 cell models of cisplatin-induced ototoxicity, avenanthramide-C, a bioactive polyphenolic alkaloid derived from Avena sativa, was associated with preservation of auditory function and outer hair cell survival, as determined by click and tone burst stimuli. This protective effects were linked to reduced ROS generation, suppression of pro-inflammatory cytokine gene expression (*il-6*, *il-1β*, and *tnf-α*), and attenuation of DNA damage signaling, supporting a multifaceted antioxidative and anti-inflammatory protective profile against cisplatin-induced cochlear injury [51]. In the context of cisplatin exposure, oxidative stress is also associated with a reduction in cellular antioxidant enzymes, while excessive ROS accumulation has been implicated in mitochondrial biogenesis dysfunction and subsequently activates apoptotic pathways [9,38,52]. Our finding further indicated that excessive ROS production led to the depletion of antioxidant enzyme-related gene expression, including *gpx*, *gstp1*, *sod1*, and *sod2*, concomitant with a decreased expression of *nrf-2*, a redox-sensitive transcription factor of oxidative stress regulatory pathway against toxic insults. So et al. emphasized the protective role of Nrf-2 through overexpression in HEI-OC1 cell, showing reduced susceptibility to cisplatin-induced cell death compared with cells expressing a transcriptionally inactive *nrf-2* mutant [53]. Our prior work has documented that *Vernonia amygdalina* enhanced *nrf-2*-mediated primary antioxidant enzyme (*cat*, *gpx*, and *sod1*) expression in cisplatin-treated transgenic zebrafish [9]. Significant insights from Jia et al. suggested that loss of Nrf-2 signaling eliminated the otoprotective effects of apigenin, highlighting the essential role of Nrf-2-drived glutamate cysteine ligase complex/GSH and SOD2 antioxidative pathway in mitigating oxidative stress-triggered hair cell death [54]. Furthermore, using in vivo and in vitro neuropathy models, Zhang et al. reported that cynarin ameliorates spinal cord injury through Nrf2-dependent NLRP3 inflammasome inhibition. The specific Nrf2/NLRP3 pathway regulation subsequently suppresses microglial pyroptosis and cellular ROS accumulation, ultimately translating into prominent functional recovery [55]. The loss of Nrf-2 displayed increased sensitivity to gentamicin-associated cochlear toxicity and early-onset hearing impairment, accompanied by spiral ganglion neuron degeneration, indicating impaired defense against frequency-specific noise exposure [56]. Collectively, the current data, consistent with previous reports, indicate that COL stem extract exerts antioxidative effects primarily via the upstream blockade of the MET channel-mediated uptake of cisplatin, coupled with the manifestation of nrf-2-regulated cytoprotective gene profile, thereby attenuating ROS overproduction and preventing hair cell apoptosis. Ototoxins exposure leads to the depletion of zebrafish lateral line neuromasts, which correlates with impaired swimming behavior since it has been used as phenotypic outcome to reflect the functional integrity of the zebrafish lateral line system [23,25]. We propose that the recovery of swimming performance represents a biomarker of hair cell protection and provides a promising rapid screening platform for otoprotectants. As predicted, cisplatin treatment significantly induced locomotor dysfunction, characterized by reduced distance moved, velocity, rotation frequency, and turn angle. In contrast, COL stem effectively ameliorates the observed functional deficits resulting from apoptotic hair cell injury. Those findings highlight the otprotective role of COL stem, which acts through antioxidant mechanisms to prevent cisplatin-induced hair cell damage.

## 5. Conclusions

**Figure 7:**
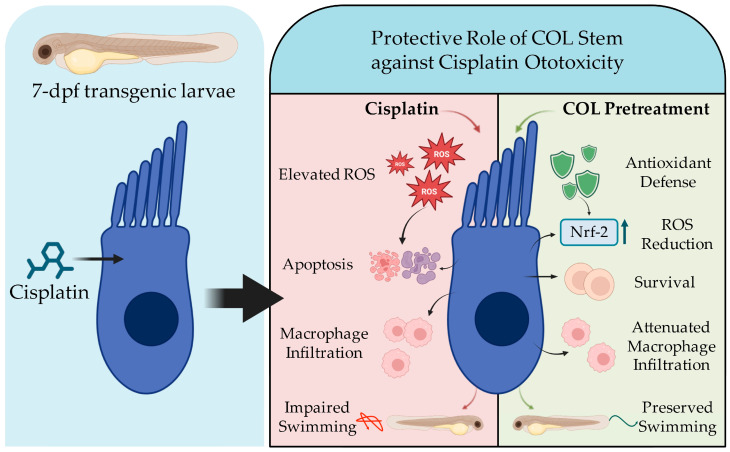
Schematic representation of the proposed otoprotective mechanisms of COL stem extract in cisplatin-induced ototoxicity. COL stem protects zebrafish lateral line hair cells from cisplatin-induced damage by preserving MET function and redox homeostasis, thereby preventing oxidative stress induced apoptosis and behavior impairment. Created in BioRender. Yang, J. (2026) https://BioRender.com/p6tlz2k.

In summary, for the first time, the present study suggests that COL stem represents a novel otoprotective agent and characterizes its protective actions against cisplatin-induced hair cell damage, and further provides insight into the underlying regulatory pathway using a tarnsgenic zebrafish-based screening platform. Our results demonstrate the otoprotective efficacy of COL stem, as it preserves later-line hair cell viability and MET functionality under cisplatin insult through ROS scavenging and regulation of nrf-2-mediated redox homeostasis, thereby attenuating oxidative–inflammatory stress and apoptosis. The analysis of zebrafish behavior phenotyping revealed a direct association between locomotor alterations and hair cell integrity, supporting its application as a high-capacity screening system (Figure 7).

## Figures and Tables

**Table 3:** Identification of major metabolites in COL stem extract.

Peak	RT (min)	Compounds	Formula	*m*/*z* (Observed)	*m*/*z* (Theoretical)	Error (ppm)	MS2 Fragments	Area	Ontology
1	0.60	Malic acid	C4H6O5	133.0142	133.0142	0.0	71, 115	1.93 × 10^9^	Organic acids
2	0.79	Citric acid	C6H8O7	191.0194	191.0197	−1.6	111, 87	1.23 × 10^9^	Organic acids
3	1.01	Succinic acid	C4H6O4	117.0190	117.0193	−2.6	73, 99	2.86 × 10^8^	Organic acids
4	1.02	Guanosine	C10H13N5O5	282.0849	282.0844	1.8	150, 133	1.90 × 10^8^	Nucleosides
5	4.93	Chlorogenic acid	C16H18O9	353.0881	353.0878	0.8	191	9.15 × 10^8^	Phenolic acids
6	5.56	Cryptochlorogenic acid	C16H18O9	353.0879	353.0878	0.3	173, 179, 135, 191	2.17 × 10^8^	Phenolic acids
7	14.08	Azelaic acid	C9H16O4	187.0976	187.0976	0.0	125, 97, 57	1.17 × 10^8^	Fatty acids
8	14.16	Cynarin	C25H24O12	515.1202	515.1195	1.4	191, 179, 353	5.00 × 10^8^	Phenolic acids

## Data Availability

The raw data supporting the conclusions of this article will be made available by the authors on request.

## Abbreviations

The following abbreviations are used in this manuscript:

COL	*Corchorus olitorius* L.
CE	catechin equivalent
dpf	day post-fertilization
DW	dry weight
ESI	electrospray ionization
GAE	gallic acid equivalent
GFP	green fluorescence protein
HCD	higher-energy collisional dissociation
UPLC-HRMS	ultra-performance liquid chromatography–high-resolution mass spectrometry
MET	mechanotransduction
MS	mass spectrometry
NR	neutral red
PLL	posterior lateral line
ROS	reactive oxygen species
TEAC	Trolox equivalent antioxidant capacity
TUNEL	terminal deoxynucleotidyl transferase dUTP nick-end labeling

## References

[1] Ivanov S.A., Zhuravskii S.G., Galagudza M.M. (2012). [Ototoxicity of cisplatin]. Vestn. Otorinolaringol..

[2] Steyger P.S. (2021). Mechanisms of Aminoglycoside- and Cisplatin-Induced Ototoxicity. Am. J. Audiol..

[3] Sternberg C.N., Skoneczna I., Kerst J.M., Albers P., Fossa S.D., Agerbaek M., Dumez H., de Santis M., Theodore C., Leahy M.G. (2015). Immediate versus deferred chemotherapy after radical cystectomy in patients with pT3-pT4 or N+ M0 urothelial carcinoma of the bladder (EORTC 30994): An intergroup, open-label, randomised phase 3 trial. Lancet Oncol..

[4] Lee D.S., Travis E.Y., Wong S.K., Collopy C., McClannahan K.S., Ortmann A.J., Rich J.T., Pipkorn P., Puram S.V., Jackson R.S. (2023). Audiologic Follow-up in Patients with Head and Neck Cancer Treated with Cisplatin and Radiation. Laryngoscope.

[5] Yang S.J., Pai J.A., Shieh M.J., Chen J.L., Chen K.C. (2023). Cisplatin-loaded gold nanoshells mediate chemo-photothermal therapy against primary and distal lung cancers growth. Biomed. Pharmacother..

[6] Wang H., Guo S., Kim S.J., Shao F., Ho J.W.K., Wong K.U., Miao Z., Hao D., Zhao M., Xu J. (2021). Cisplatin prevents breast cancer metastasis through blocking early EMT and retards cancer growth together with paclitaxel. Theranostics.

[7] Guo J., Chai R., Li H., Sun S. (2019). Protection of Hair Cells from Ototoxic Drug-Induced Hearing Loss. Hearing Loss: Mechanisms, Prevention and Cure.

[8] Wu P., Wu X., Zhang C., Chen X., Huang Y., Li H. (2021). Hair Cell Protection from Ototoxic Drugs. Neural Plast..

[9] Cheng H., Wu Y., Yang J. (2025). Vernonia amygdalina displays otoprotective effects via antioxidant pathway on cisplatin-induced hair cell loss in zebrafish. Arch. Toxicol..

[10] Loumerem M., Alercia A. (2016). Descriptors for jute (*Corchorus olitorius* L.). Genet. Resour. Crop Evol..

[11] Ahmed D.A., Slima D.F. (2018). Heavy metal accumulation by *Corchorus olitorius* L. irrigated with wastewater. Environ. Sci. Pollut. Res..

[12] Yang Z., Wu Y., Dai Z., Chen X., Wangb H., Yang S., Xie D., Tang Q., Cheng C., Xu Y. (2020). Comprehensive transcriptome analysis and tissue-specific profiling of gene expression in jute (*Corchorus olitorius* L.). Ind. Crops Prod..

[13] Abdel-Razek M.A.M., Abdelwahab M.F., Abdelmohsen U.R., Hamed A.N.E. (2022). Pharmacological and phytochemical biodiversity of *Corchorus olitorius*. RSC Adv..

[14] Ncube B., Dlamini B., Beswa D. (2022). Effect of Common Cooking and Drying Methods on Phytochemical and Antioxidant Properties of *Corchorus olitorius* Identified Using Liquid Chromatography-Mass Spectrometry (LC-MS). Molecules.

[15] Lee K., Chung Y. (2025). Anti-Inflammation and Anti-Hypersensitivity Effects of *Corchorus olitorius* Extract in Cellular and NC/Nga Mouse Models of Atopic Dermatitis. Prev. Nutr. Food Sci..

[16] Biswas A., Dey S., Huang S., Deng Y., Birhanie Z.M., Zhang J., Akhter D., Liu L., Li D. (2022). A Comprehensive Review of C. capsularis and *C. olitorius*: A Source of Nutrition, Essential Phytoconstituents and Pharmacological Activities. Antioxidants.

[17] Biswas A., Dey S., Xiao A., Huang S., Birhanie Z.M., Deng Y., Liu L., Li D. (2023). Phytochemical content and antioxidant activity of different anatomical parts of *Corchorus olitorius* and *C. capsularis* during different phenological stages. Heliyon.

[18] Azuma K., Nakayama M., Koshioka M., Ippoushi K., Yamaguchi Y., Kohata K., Yamauchi Y., Ito H., Higashio H. (1999). Phenolic antioxidants from the leaves of *Corchorus olitorius* L. *J. Agric*. Food Chem..

[19] Pramanik P.K., Chakraborti S., Bagchi A., Chakraborti T. (2020). Bioassay-based *Corchorus capsularis* L. leaf-derived beta-sitosterol exerts antileishmanial effects against *Leishmania donovani* by targeting trypanothione reductase. Sci. Rep..

[20] Wagdy R., Abdelkader R.M., El-Khatib A.H., Linscheid M.W., Hamdi N., Handoussa H. (2019). Neuromodulatory Activity of Dietary Phenolics Derived from *Corchorus olitorius* L. *J*. Food Sci..

[21] Zakaria Z.A., Safarul M., Valsala R., Sulaiman M.R., Fatimah C.A., Somchit M.N., Mat Jais A.M. (2005). The influences of temperature and naloxone on the antinociceptive activity of *Corchorus olitorius* L. in mice. Naunyn Schmiedeberg’s Arch. Pharmacol..

[22] Hung T.H., Cheng C.P., Chiou W.C., Hsia T.L., Liu H.K., Lu H.F., Lai Y.H., Tsai S.T., Chen C.J., Huang C. (2026). Longan flower water extract promotes sleep in mice via the serotonin-melatonin axis. J. Sci. Food Agric..

[23] Cheng H.L., Lee S.C., Chang-Chien J., Su T.R., Yang J.J., Su C.C. (2023). Protective mechanism of ferulic acid against neomycin-induced ototoxicity in zebrafish. Environ. Toxicol..

[24] Hsu C.-F., Yang F.-A., Liu S.-C., Li S.-Y., Song H.-W., Liang Z.-C., Yang J.-J. (2020). Protective Role of Vernonia Amygdalina Leaf Aqueous Extract Against Neomycin-Induced Hair Cell Damage in Transgenic Zebrafish. Curr. Top. Nutraceutical Res..

[25] Lai T.W., Cheng H.L., Su T.R., Yang J.J., Su C.C. (2022). Cichoric Acid May Play a Role in Protecting Hair Cells from Ototoxic Drugs. Int. J. Mol. Sci..

[26] Zakaria Z.A., Ghani Z.D., Nor R.N., Gopalan H.K., Sulaiman M.R., Jais A.M., Somchit M.N., Kader A.A., Ripin J. (2008). Antinociceptive, anti-inflammatory, and antipyretic properties of an aqueous extract of *Dicranopteris linearis* leaves in experimental animal models. J. Nat. Med..

[27] Mokgalaboni K., Phoswa W.N. (2023). *Corchorus olitorius* extract exhibit anti-hyperglycemic and anti-inflammatory properties in rodent models of obesity and diabetes mellitus. Front. Nutr..

[28] El-Tohory S., Zeng W., Huang J., Moussa M.G., Dong L., Ismael M.A., Khalifa O., Salama M.A., Hekal M.A., Basyouny M.A.E. (2024). Effect of intercropping and biochar amendments on lead removal capacity by *Corchorus olitorius* and *Zea mays*. Environ. Sci. Pollut. Res. Int..

[29] Mukul M.M., Akter N. (2021). Morpho-anatomical variability, principal component analysis and Euclidean clustering of tossa jute (*Corchorus olitorius* L.). Heliyon.

[30] Jha D.K., Parida S., Pradhan S., Dey N., Majumder S. (2025). Genome-wide analysis of the laccase gene family in tossa jute (*Corchorus olitorius*): Insights into stem development, lignification, and responses to abiotic stress. Front. Plant Sci..

[31] Hassan A.Z., Mekhael M.K., Hanna A.G., Simon A., Tóth G., Duddeck H. (2019). Phytochemical investigation of *Corchorus olitorius* and *Corchorus capsularis* (Family Tiliaceae) that grow in Egypt. Egypt. Pharm. J..

[32] Ali M.R., Ibrahim H.H., Salah-Eldin A.A. (2024). Unveiling the Chemical Composition, Bioactive Profile and Antioxidant Capacity of Dried Egyptian Jew’s Mallow Stems as a Promising Anticancer Agent. Molecules.

[33] Duke J.A. (1983). Handbook of Energy Crops.

[34] Im G.J., Chang J.W., Choi J., Chae S.W., Ko E.J., Jung H.H. (2010). Protective effect of Korean red ginseng extract on cisplatin ototoxicity in HEI-OC1 auditory cells. Phytother. Res..

[35] Choi J., Im G.J., Chang J., Chae S.W., Lee S.H., Kwon S.Y., Chung A.Y., Park H.C., Jung H.H. (2013). Protective effects of apocynin on cisplatin-induced ototoxicity in an auditory cell line and in zebrafish. J. Appl. Toxicol..

[36] Thomas A.J., Wu P., Raible D.W., Rubel E.W., Simon J.A., Ou H.C. (2015). Identification of small molecule inhibitors of cisplatin-induced hair cell death: Results of a 10,000 compound screen in the zebrafish lateral line. Otol. Neurotol..

[37] Gale J.E., Marcotti W., Kennedy H.J., Kros C.J., Richardson G.P. (2001). FM1-43 dye behaves as a permeant blocker of the hair-cell mechanotransducer channel. J. Neurosci..

[38] Lee D.S., Schrader A., Zou J., Ang W.H., Warchol M., Sheets L. (2024). Cisplatin drives mitochondrial dysregulation in sensory hair cells. bioRxiv.

[39] Lee D.S., Schrader A., Zou J., Ang W.H., Warchol M.E., Sheets L. (2024). Direct targeting of mitochondria by cisplatin leads to cytotoxicity in zebrafish lateral-line hair cells. iScience.

[40] Ouyang L., Ma L., Feng Y. (2025). Protective effects of MET channels on aminoglycosides- and cisplatin-induced ototoxicity. Int. J. Med. Sci..

[41] Ramkumar V., Mukherjea D., Dhukhwa A., Rybak L.P. (2021). Oxidative Stress and Inflammation Caused by Cisplatin Ototoxicity. Antioxidants.

[42] Li M., Liu J., Liu D., Duan X., Zhang Q., Wang D., Zheng Q., Bai X., Lu Z. (2021). Naringin attenuates cisplatin- and aminoglycoside-induced hair cell injury in the zebrafish lateral line via multiple pathways. J. Cell Mol. Med..

[43] Lim K.H., Park S., Han E., Baek H.W., Hyun K., Hong S., Kim H.J., Lee Y., Rah Y.C., Choi J. (2024). Protective Effects of Fasudil Against Cisplatin-Induced Ototoxicity in Zebrafish: An In Vivo Study. Int. J. Mol. Sci..

[44] Tan W.J.T., Song L. (2023). Role of mitochondrial dysfunction and oxidative stress in sensorineural hearing loss. Hear. Res..

[45] Yuan Y., Yuan L., Yang J., Liu F., Liu S., Li L., Liao G., Tang X., Cheng J., Liu J. (2024). Autophagy-deficient macrophages exacerbate cisplatin-induced mitochondrial dysfunction and kidney injury via miR-195a-5p-SIRT3 axis. Nat. Commun..

[46] Zhang N., Cai J., Xu L., Wang H., Liu W. (2020). Cisplatin-Induced Stria Vascularis Damage Is Associated with Inflammation and Fibrosis. Neural Plast..

[47] Zhao X.L., Yu L., Zhang S.D., Ping K., Ni H.Y., Qin X.Y., Zhao C.J., Wang W., Efferth T., Fu Y.J. (2020). Cryptochlorogenic acid attenuates LPS-induced inflammatory response and oxidative stress via upregulation of the Nrf2/HO-1 signaling pathway in RAW 264.7 macrophages. Int. Immunopharmacol..

[48] Lin Y., Li L., Cheng J. (2025). Chlorogenic Acid Improves Salicylic Acid-Induced Cochlear Injury by Reducing Oxidative Stress and Inflammatory Response Through TLR4/NF-kappaB Signalling Pathway. Clin. Exp. Pharmacol. Physiol..

[49] Borse V., Al Aameri R.F.H., Sheehan K., Sheth S., Kaur T., Mukherjea D., Tupal S., Lowy M., Ghosh S., Dhukhwa A. (2017). Epigallocatechin-3-gallate, a prototypic chemopreventative agent for protection against cisplatin-based ototoxicity. Cell Death Dis..

[50] Rahman M.T., Bailey E.M., Gansemer B.M., Pieper A.A., Manak J.R., Green S.H. (2023). Anti-inflammatory Therapy Protects Spiral Ganglion Neurons After Aminoglycoside Antibiotic-Induced Hair Cell Loss. Neurotherapeutics.

[51] Umugire A., Nam Y.S., Nam Y.E., Choi Y.M., Choi S.M., Lee S., Cho J.H., Cho H.H. (2023). Protective Effect of Avenanthramide-C on Auditory Hair Cells against Oxidative Stress, Inflammatory Cytokines, and DNA Damage in Cisplatin-Induced Ototoxicity. Int. J. Mol. Sci..

[52] Ficek K., Stepien-Slodkowska M., Kaczmarczyk M., Maciejewska-Karlowska A., Sawczuk M., Cholewinski J., Leonska-Duniec A., Zarebska A., Cieszczyk P., Zmijewski P. (2014). Does the A9285G Polymorphism in Collagen Type XII alpha1 Gene Associate with the Risk of Anterior Cruciate Ligament Ruptures? *Balk*. J. Med. Genet..

[53] So H.S., Kim H.J., Lee J.H., Lee J.H., Park S.Y., Park C., Kim Y.H., Kim J.K., Lee K.M., Kim K.S. (2006). Flunarizine induces Nrf2-mediated transcriptional activation of heme oxygenase-1 in protection of auditory cells from cisplatin. Cell Death Differ..

[54] Jia G., Mao H., Zhang Y., Ni Y., Chen Y. (2022). Apigenin alleviates neomycin-induced oxidative damage via the Nrf2 signaling pathway in cochlear hair cells. Front. Med..

[55] Zhang B., Yu J., Bao L., Feng D., Qin Y., Fan D., Hong X., Chen Y. (2024). Cynarin inhibits microglia-induced pyroptosis and neuroinflammation via Nrf2/ROS/NLRP3 axis after spinal cord injury. Inflamm. Res..

[56] Honkura Y., Matsuo H., Murakami S., Sakiyama M., Mizutari K., Shiotani A., Yamamoto M., Morita I., Shinomiya N., Kawase T. (2016). NRF2 Is a Key Target for Prevention of Noise-Induced Hearing Loss by Reducing Oxidative Damage of Cochlea. Sci. Rep..

